# Safely balancing a double-edged blade: identifying and mitigating emerging biosecurity risks in precision medicine

**DOI:** 10.3389/fmed.2024.1364703

**Published:** 2024-03-20

**Authors:** Diane DiEuliis, James J. Giordano

**Affiliations:** ^1^Center for the Study of Weapons of Mass Destruction, National Defense University, Washington, DC, United States; ^2^Departments of Neurology and Biochemistry, Georgetown University Medical Center, Washington, DC, United States; ^3^Defense Medical Ethics Center, Uniformed Services University of the Health Sciences, Bethesda, MD, United States; ^4^Simon Center for the Professional Military Ethic, United States Military Academy, West Point, NY, United States

**Keywords:** precision medicine, biodata, bioweapons, cyberbiosecurity, biodefense

## Abstract

Tools and methods of precision medicine are developing rapidly, through both iterative discoveries enabled by innovations in biomedical research (e.g., genome editing, synthetic biology, bioengineered devices). These are strengthened by advancements in information technology and the increasing body of data—as assimilated, analyzed, and made accessible—and affectable—through current and emerging cyber—and systems- technologies. Taken together, these approaches afford ever greater volume and availability of individual and collective human data. Machine learning and/or artificial intelligence approaches are broadening this dual use risk; and in the aftermath of COVID-19, there is growing incentive and impetus to gather more biological data from individuals and their environments on a routine basis. By engaging these data—and the interventions that are based upon them, precision medicine offer promise of highly individualized treatments for disease and injury, optimization of structure and function, and concomitantly, the potential for (mis) using data to incur harm. This double-edged blade of benefit and risk obligates the need to safeguard human data from purloinment, through systems, guidelines and policies of a novel discipline, cyberbiosecurity, which, as coupled to ethical precepts, aims to protect human privacy, agency, and safety in ways that remain apace with scientific and technological advances in biomedicine. Herein, current capabilities and trajectories precision medicine are described as relevant to their dual use potential, and approaches to biodata security (*viz.*- cyberbiosecurity) are proposed and discussed.

## Introduction

In this Perspective, we summarize salient highlights from our and others’ prior work to provide background and context related to issues of dual use biotechnology, biosecurity, and ethics relevant to precision medicine. The earlier term, “personalized medicine,” i.e., the aspiration to individualize therapeutics based upon patients’ unique characteristics and/or needs, has now ceded to capabilities that are far more data- driven in their specificity and precision. To wit, current practices of precision medicine now entail assessments and treatments that focus upon idiosyncratic (and key collective) genotypic vulnerabilities, phenotypic expressions, environmental exposures, and personal and familial medical and meta-information (1).

The abilities to acquire, assimilate, synthesize, and analyze these diverse and vast specific data have been enabled by advances in information (i.e., data and cyber) science and technology (2). Iterative developments, and integrative convergence of big data, machine learning and artificial intelligence tools and techniques have progressed the speed, effectiveness, and efficiency of metadata analytics. The ability to conjoin genotypic, phenotypic, ecological, and situational (bio-psychosocial) data enables individually-specific interventions that are expanding the palette of viable medical approaches (3). As well, the ability to sequence, create, and/or manipulate genes with precision has transformed the facility and scope of biomedical research capability and the range of translational applications of research findings and deliverables. A prime example of such tools-to-task throughput is the extent to which CRISPR-techniques have allowed more detailed understanding of genetic function, rapid creation of animal models of pathology, and genetically modified and personalized treatments (4). Yet, while these advances offer definable benefits to health and wellness, they also can pose risks fostered by various trajectories of potential misuse (5). Thus, we opine the need to clearly and realistically define, acknowledge, and foster preparedness for benefits, burdens, risks and threats—as well as the ethical issues posed by current and emerging precision medicine toolkit. Our aim is to both generate greater awareness, and prompt ongoing attention to developing prudent norms for the conduct of precision medicine research and uses-in-practice.

## Data drive precision medicine and its anticipated benefits—and risks

The progressive shift to more rigorous precision medicine is driven by individuals’ biological data (i.e., biodata), which are becoming more ubiquitously available (6). The relative ease and reduced costs of genome sequencing allow individuals to rather inexpensively purchase their own genomic data from direct-to-consumer providers such as *Ancestry* or *23&me*. Of note is that the low costs of the tests are due to the fact that these companies can sell such data to third party purchasers that are interested in developing precision diagnostics and/or interventions. Many individuals who purchase such tests are unaware that their genomic data may be used beyond their original intent and consent.

There are pros and cons to increasing insight to individuals’ and collective genotypic patterns. Knowledge of a specific gene identified as causative for a certain cancer, for example, may allow early medical evaluation and treatment, and may be important to define environmental and lifestyle risks that could be modified for health promotion. Exemplary of this, defined genetic profiles for types of breast cancer(s) are used to generate and provide tailored evaluative and treatment protocols with proven success in contributing to patient survivability. However, many pathologies are multi-genomic and have complex co-morbidities. In these cases, genotypic information is often not directly beneficial for therapeutics, at least at present. Thus, the utility of genomic research and health information technology (HIT) to the routine practice of medicine remains a work-in-progress. Yet, in the interim, human biodata can be applied other arenas, including those that could confer harm (7). This prompts questions of (1) whether and to what extent such information is individually useful (and valuable); (2) if and how this information can and should be proactively used for more communitarian public health efforts (e.g., toward enhanced understanding of patho-etiologies, mechanisms, and eventual development of preventative and/or treatment approaches); and (3) the degree—and processes—by which this information should be protected, and/or made available for other use(s). This latter point brings into stark relief the issue of dual-use, which, while formally defined as the viability and application of a tool or technique for more than one intent or purpose, has become more contextually applied to refer to the use of biomedical methods and technologies in ways that could incur disruptive, harmful effects (i.e., dual use research of concerns; DURC) (8).

For instance, during the COVID pandemic, advancements in biotechnology enabled concomitant viral testing for SARSCoV2 sequences in patients and whole genome screening (9). To be sure, there are indisputable advantages conferred by this approach: those who may be uniquely genetically susceptible to COVID could be rapidly identified and advised to use greater social distancing or other protective measures; while those at little identified risk could continue to maintain more relaxed social engagement. But information is not socially neutral; rather, it is interpreted, evokes meaning(s), and fosters sentiments that affect inter-personal and collective regard and treatment (10). Thus, ethical questions arise regarding the stigmatization of those characterized in particular ways; and if and how such stigma could influence public attitudes, conduct, and healthcare policy and practices.

There are additional concerns beyond ethical consideration of attitudinal bias, differential regard, and distributional asymmetries. Genomic and/or other biodata can also be used for disruptive—if not destructive—effect (7). Knowledge of genetic vulnerabilities could make viable access to and mechanisms of individual and collective harm. As more is learned about (human, animal, and plant) genetic susceptibilities to pathogens and pathogenic adaptations, there is an increased chance, if not strong probability, that this information could be utilized for two ways of leveraging of power on a variety of scales. First, these data may be corrupted and altered in ways that depict individuals and/or groups as having a certain trait, condition or disorder—or lack thereof—which would impact their access or inaccess to employment, healthcare resources, and medical treatments (11). And while some legal protection may be provided by certain accords (e.g., the Genetic Information Non-discrimination Act; GINA), this does not counter established requirements and/or disqualifications for particular jobs, insurance, and modes of care that are dependent upon defined characteristics and medical conditions. Thus, misinformation could lead to mistreatment in a number of dimensions (12).

Second, just as genetic and phenotypic information can be used to craft precision therapeutics, it can also be employed to develop “precision pathogens” based upon individual or group sensitivities and susceptibilities (1). Importantly, the “scope of precision,” that is, the degree of desired effect needs only to be relevant to (1) the accuracy of affecting the intended target; (2) the “positive” induction of the intended disruptive action—i.e., the success of the pathologic outcome; and/or (3) the combinatory value of both (i.e., a “hit” versus a “miss” as regards both target and influence). The likelihood of true negative effect(s) need not be as stringent, given that any disruptive “side effects” could be regarded as tolerable, if not desirable.

**Figure 1:**
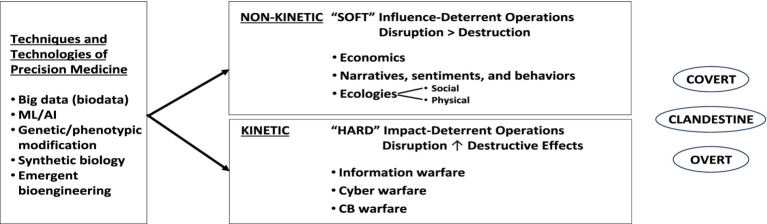
Diagrammatic illustration of those ways that precision medicine approaches (e.g., biodata, coupled to machine learning/AI and current and emerging tools of genetic assessment, editing, synthetic biology, and bioengineering) could be utilized in non-kinetic and/or kinetic engagements/ways to affect individuals and groups, either covertly, clandestinely, or overtly (see text for details).

If we consider “weapons,” as literally defined, to be “ways of contending against others” (13), then as shown in Figure 1, these approaches can be utilized for disruptive economic and ecologic effect (i.e., non-kinetically, as “soft weapons”) to exercise financial hegemony in local, regional and/or global markets (14). Alternatively, they could be employed for disruptive, destructive effect (i.e., kinetically, as “hard weapons”) to yield power through more profound articulation of specifically individual [viz., high profile target (s)] or mass casualty [e.g., morbidity or mortality, actions, (15)].


Ml/AI=machinelearning/artificialintelligence

CB=chembio

## Protecting biodata from misuse

In light of the iterative elucidation of, and ability to target genetic bases of diseases; and the disruptive/destructive dual-use possibilities enabled by such uses of biodata and preventive medical approaches, some balance must be struck. This is easier said than done. The research community has been pressed to share biodata in a number of public databases, such as *Genbank,* which aim to stimulate broader discovery and maximize value from taxpayer investments in biomedicine (16). It should also be noted that healthcare and precision medicine are not the only arenas in which human biodata are being gathered and used. In the aftermath of COVID, and with the greater ease and reduced cost of available technology, DNA sequencing is being performed in a variety of environments, inclusive of buildings, air filters, wastewater, and natural ecosystems. These sequencing activities indisputably include human data which can be identifiable (17). To what ends might this information be employed?

We have discussed the risks of misuse of publicly available pathogen data in other publications (11, 18), and believe that human genomic and phenotypic data occupy a distinct category of consideration for enhanced protections. While individual patient privacy as has always been primary, there is now very real—and increasing—risk of such data being used to incur targeted physical harm (7). Prior to the era of precision medicine,” de-identifying” patient data involved a fairly simple process of obfuscating key informational indicators. But as biodata expand to include genetic information, imaging data, facial recognition, and behavioral traits, the ability to “re-identify” individuals by employing big data and machine learning systems and methods of pattern recognition, generation, and assignation is increasingly possible, and ever more facile (19).

Moreover, the protections of human subjects and patients (in the United States) provided by The Common Rule (20) and HIPAA (Health Insurance Portability and Accountability Act of 1996) do not explicitly afford encryption or security of human genomic data collected during research; nor do they govern the development and use of data and cyber technologies that can be employed to identify patients from existing data sets. Although HIPAA governs a variety of characteristics of personally identifiable information (PII)—excluding human genomic data—these protections are only applicable (and enforceable) within the US, and therefore risks and threats may be generated within multinational partnerships that engage US institutions’ biodata for enterprises conducted outside US borders.

## Further—and better—defining the risk and threat space(s)

The intersection of biodata and information systems establishes a special niche of biosecurity consideration, increasingly recognized as “cyberbiosecurity (21), or “digital biosecurity” (22). As we have explicated, although being based upon fundamental principles and methods of cybersecurity in general, “cyberbiosecurity” represents a somewhat unique domain in that it addresses a “novel sphere of hazards that surrounds the generation, use, and misuse of biodata” (7, 23).

Cyberbiosecurity approaches aim to identify such risks and hazards by mapping some of the intricate relationships that exist between computational and experimental workflows in biotechnology. Awareness of the need for cyberbiosecurity is growing, but the breadth and complexity of data used across different contexts have made structured policy and governance difficult. Institutions that gather such data are varied, and include academia, biomedical industries and laboratories, non-profits, health care institutions, hospitals and the government. As well, there is considerable variability in the biodata user base: biologists, physicians, engineers, physicists, software designers, manufacturers, etc., each and all of whom may have differing awareness of cyberbiosecurity risks (or, even traditional biosecurity risks).

Currently, there are efforts underway to create cyberbiosecurity solutions (24). Essential to any such enterprise is renewed dedication to improved, diligent cyber hygiene. The number of healthcare data breaches due to hacking is concerning in that it has incurred millions of dollars of loss to healthcare insurers, and has posed demonstrable risk—and realistic threat- to patient privacy and safety. At the Federal level, a Health Information Sharing and Analysis Center (H-ISAC) has been established so that best practices can be shared across the healthcare community,^1^ and membership in the H-ISAC should be encouraged. However, specific issues related to the risks of genomic data, as described herein, require pragmatic and prudent asset (*viz.*- systems) and policy solutions to assure sustainable data protection. The National Institute of Standards and Technology (NIST) has taken positive steps in this direction by building tools for genomic cybersecurity that are available at no cost (25).

There have been instances of cybersecurity breaches and various forms of hacking (26), testifying to the desires of bad actors to access and corrupt human biodata. Fortunately, frank misuse of biodata resulting in human harms has not yet occurred on an appreciable scale. The use of machine learning/AI provides a greater depth of understanding beyond genomic databases; an AI tool trained on a publicly available chemical database has been used to formulaically generate possible chemical and/or biological weapons (27). Similarly, a Large Language Model (LLM) confirmed the rapidity with which pathogen engineering information can be acquired (28). Other studies note that AI/ML does not confer information that goes statistically beyond that which could be gathered from scraping the internet or using publicly available genetic databases (29)—however it does afford the ability to get that information with greater rapidity and ease. While current policy addressing Dual Use Research of Concern (DURC) identifies “knowledge and information”—implicitly inclusive of biodata—as a risk/threat category of in its definition, it is of limited focus and scope, and is restricted to only particular agents and pathogens.

A valuable resource to more broadly construed cyberbiosecurity risks and threats, such as those discussed above, may be the DURC “Companion Guide” (HHS),^2^ which might offer viable direction for managing data that could be employed in disruptive and/or destructive ways. However, these guidelines are not mandatorily applicable to commercial entities using biodata and manufacturing chembio-agents. While many companies enforce oversight and regulation of agents and substances that are similar to those that have been identified as dangerous, such regulatory action remains discretionary, which, when taken together with the somewhat narrow scope of categorization of dangerous substances provided by DURC policies and regnant treaties and conventions, incur significant limitations—and weaknesses—to these current practices, as evidenced by recent statements made by the National Science Advisory Board for Biosecurity (NSABB) (30).

## Discussion

Important steps for developing and implementing the types of cyberbiosecurity tools and methods that will be required to meet the evolving capabilities of emergent science and technology are (1) accurate forecasting (*viz.*- prediction) of the scientific and technological landscape as relevant to possible and probable risks and threats; and (2) foresight (*viz.*- preparative planning and articulation) to most promptly and aptly address these current and evolving risks/threats. We have posited, and re-assert here that emerging biotechnologies—such as biodata systems—demand equivalent (if not equal) dedication to development of ethically-informed policies to guide and govern their use (s)-in-practice (31, 32).

To engage such tasks, we have described a general paradigm to assess and mitigate risks posed by emerging biotechnologies, inclusive of those developed and employed for biodata acquisition, assessment and use (33–35). As shown in Table 1, this paradigm entails basic responsibilities, poses essential questions, frames key contingencies, and offers directives for guidelines and policies.

**Table 1:** Domains and dimensions of a proposed risk assessment and mitigation paradigm for emerging bioscience and technologies (S/T = science and technology).

6-W questions	As framed by… 6-C considerations
*What* S/T are available for current use? *Why* is S/T considered or advocated for use? *Who* will receive S/T? When will S/T be considered (algorithm/protocol)? *Where* will S/T be engaged (e.g., locale, settings, etc.)? *Which* mechanisms will be in place for ongoing provisions of services/resources required for safety/security of S/T use?	*Capacities* and limitations of the S/T *Consequences* incurred by S/T on individuals and systems in the short, intermediate, and long-term *Character* of the use case and user(s) affected by S/T *Contexts* of need and value that influence use of S/T *Continuity* of research and revision of guidance principles and policy *Consent or Consensus* for use based upon provision of most information possible

To be effective and operationalized within a larger context of multi-national research, development and use of precision medicine approaches should address several core policy and ethical questions.

- How should unrestricted access to, and/or “tampering” with biological data be regulated by international law?
- Which mechanism (s) must be established—and ratified—for global enforcement?
- Should the Biological and Toxins Weapons Convention (BTWC) and/or Chemical Weapons Convention (CWC) consider uses and/or compromises to biodata as contributors toward chem-bio weapon development? And if so, how might these conventions be amended to address the techniques, technologies, and use-cases that are regarded to be a risk or threat? (36).

As we have noted, mitigation or prevention of risks has both challenges and opportunities, and a unitary approach will be unlikely to afford an effective solution in either domain. In contrast, we offer that a set of approaches, if used in concert, would have higher utility. We are optimistic about international best practices that are being created for other types of data (such as those proposed for financial data), which could offer a template for effective biodata security. But it will be critical to recognize ways that multinational entities’ differing cultures and perspectives contribute to the economic, proprietary, ethical and legal bases of their programs in scientific and technological research, development and uses-in-practice. Further, it must be understood how these variables factor in realistic assessment (and enterprise) of both cooperation and competition.

Given multi-national (i.e., globally relevant) and more locally particular (e.g., region specific) development and application (s) of biodata, the discourse should include each and all of the relevant stake- and share-holders involved. It is likely that current international standards for biodata use will need to be revisited, revised, and/or more realistic—and meaningful—norms and guidelines established anew, as pertinent to the possibilities for such data—and data-derived products (e.g., chemical and biological agents) to be non-kinetically and/or kinetically employed toward disruptive and destructive ends.

In conclusion, the current pace and palette of progress in the biosciences enable ever greater power to modify existing and generate new organisms, and improve treatment (s) for an expanding number of diseases. Yet, at the same time, such advances can impact the biosecurity of individuals and collectives, and give rise to questions of how the risks and threats they pose can and should be evaluated and addressed. Such considerations and concerns undergird our evocation for improved risk assessment, and formulation of tools and methods for (1) this focus and depth of inquiry, and (2) the oversight and governance of products and capabilities that such research and development may yield. It is to these tasks and goals that our group remains dedicated and diligent.

## Data availability statement

The original contributions presented in the study are included in the article/supplementary material, further inquiries can be directed to the corresponding author.

## Author contributions

DD: Conceptualization, Data curation, Formal analysis, Funding acquisition, Investigation, Methodology, Project administration, Resources, Software, Supervision, Validation, Visualization, Writing – original draft, Writing – review & editing. JG: Conceptualization, Data curation, Formal analysis, Funding acquisition, Investigation, Methodology, Project administration, Resources, Software, Supervision, Validation, Visualization, Writing – original draft, Writing – review & editing.

## Footnotes

^1^ https://h-isac.org/

^2^ https://www.phe.gov/s3/dualuse/documents/durc-companion-guide.pdf
